# Re-imagining Reproduction: The Queer Possibilities of Plants

**DOI:** 10.1093/icb/icad012

**Published:** 2023-04-06

**Authors:** Banu Subramaniam, Madelaine Bartlett

**Affiliations:** Department of Women, Gender, Sexuality Studies, UMass Amherst, 130 Hicks Way, Amherst, MA 01003, USA; Department of Biology, UMass Amherst, 611 N Pleasant St, Amherst, MA 01003, USA

## Abstract

How did plant sexuality come to so hauntingly resemble human sexual formations? How did plant biology come to theorize plant sexuality with binary formulations of male/female, sex/gender, sperm/egg, active males and passive females—all of which resemble western categories of sex, gender, and sexuality? Tracing the extant language of sex and sexuality in plant reproductive biology, we examine the histories of science to explore how plant reproductive biology emerged historically from formations of colonial racial and sexual politics and how evolutionary biology was premised on the imaginations of racialized heterosexual romance. Drawing on key examples, the paper aims to (un)read plant sexuality and sexual anatomy and bodies to imagine new possibilities for plant sex, sexualities, and their relationalities. In short, plant sex and sexuality are not two different objects of inquiry but are intimately related—it is their inter-relation that is the focus of this essay. One of the key impulses from the humanities that we bring to this essay is a careful consideration of how terms and terminologies are related to each other historically and culturally. In anthropomorphizing plants, if plant sexuality were modeled on human sexual formations, might a re-imagination of plant sexuality open new vistas for the biological sciences? While our definitions of plant sexuality will always be informed by contemporary society and culture, interrogating the histories of our theories and terminologies can help us reimagine a biology that allows for new and more accurate understandings of plants, plant biology, and the evolution of reproduction.

A striking aspect of contemporary biology is the haunting similarity in the vocabulary between the reproductive biology of plants and that of humans. Our biological theories of sexuality have been scaffolded onto key frameworks, theories, and terminologies. For example, in discussing plants and humans, we use the same categories of “male” and “female,” and concepts of “sex” and “gender.” We talk about male and female plants, male and female flowers, and male and female parts of flowers. Sex, as a term, is also complex because it encompasses so much of the biological landscape. We talk about sex in multiple registers: As a noun, sex represents a particular kind of body, but sex is also an act; as an adjective, sexual demarcates certain anatomy, physiology, and behaviors as exclusively sexual or reproductive; and as a verb, to sex an object is the ability to assign it a sexual type (most often binary male and female). How did this come to be? Why did plants, whose biologies—morphologies, anatomies, development, physiology, ecologies, and life histories—differ so greatly from humans, come to be made in the image of humans?

In this essay, we bring together insights from the fields of feminist science and technology studies (fSTS) and plant reproductive biology to explore how theories of reproduction traveled across fields and lay out their consequences. At the heart of our analysis is the critical insight from fSTS that the realms of nature/culture and science/society are not binary opposites. Instead, ideas, theories, and frameworks travel from social worlds to natural worlds and then back again in a loop of intellectual and cultural exchange. Categories such as sex, gender, race, and sexuality are simultaneously biological and cultural categories. Take a category like race. It is not universal or stable; it has changed and mutated over time. Race is not a singular character or a variable; it is dynamic and best understood as an evolving “racial formation” (Omi and Winant 2014). While beyond the scope of this essay, historians have tracked how old racial logics persist in contemporary biological frameworks on population (Grene 1990; Weasel 2022); and current work on the genetics of health (Roberts 2014; Hammonds and Reverby 2019; Pollock et al. 2021); and ancestry (Wailoo et al. 2012; Shim et al. 2015; Hamilton 2020). Race has different formations across the world and changes over time as civil rights and other social movements challenge the racist underpinnings of the world. For example, the Irish, once pathologized in the United States, are now seen as white (Ignatiev 1994). Similar stories span the biological sciences. In short, there is no pure nature or culture, only naturecultures (Haraway and Goodeve 2013). Naturecultures as a concept exemplifies the case of plant sex and sexuality. In this essay, we show that plant reproductive biology, while ostensibly about plants (read nature), is really narrating a story of western colonial sexuality (read culture). Thinking nature-culturally reveals the entanglements of nature and cultures, reminding us to always locate biological theories within their histories.

In this essay, we track ideas and theories about reproduction through the history of science. We begin with a brief history of how plants came to acquire sex based on western conceptions of sexuality and discuss why this is important. We then turn to the astonishing diversity of plant worlds and the complexities of plant reproduction to argue that too much is lost when we shoe-horn plants into inappropriate and inaccurate categories of western sexuality. We explore the many distinctions and innovations in plant biology that are erased in this facile comparison. It obscures and limits our understanding of plant biology and plant diversity. We end with suggestions for alternate vocabularies and terminologies for re-imagining plant reproductive biology. We should state at the outset that our goal is to better represent—in botanical vocabulary and theories—the biology of plants. While we urge a move away from human-centered language and frameworks, we are not contesting the well-established and foundational processes outlined by plant biology.

## Why theories of sex, gender, and sexuality matter

Why should we care if we talk about plants and animals in human terms? In recent years, key scholars have argued that there is no universal “human” or a “monolithic” society but rather particular humans within stratified societies (Wirth 2022). Historians have demonstrated the devasting impact of hierarchies of sex, gender, sexuality, race, and nation, each of which has spawned centuries of oppression (Collins 1990). These are not innocent categories. In each, some humans are considered superior to others. Science is at the heart of the story (Subramaniam 2014). For example, scientific theories deemed women in western countries as lacking rationality (Plumwood 1993), and therefore (even white women) were denied the right to be independent, vote, run for political office, or have access to higher education. The doctrine of coverture under Anglo-Saxon law erased women’s independent existence, so once she married, she lost her rights, and her children and she were deemed the property of her husband (Zaher 2002). Women in the US gained the right to open a bank account as recently as the 1960s, and the Equal Credit Opportunity Act that prohibited credit discrimination based on gender was only passed in 1974. The logics that underlay discrimination were firmly grounded in scientific theories of sex and gender.

Racial hierarchies are at the heart of the brutalities under slavery and the logic of colonialism. The concept of race emerged and remains a potent category (Smedley and Smedley 2005). Colonized and enslaved people were deemed inferior in their biologies—anatomy, physiology, and evolution. As Dorothy Roberts (2014) argues, race was invented as an instrument to promote racism, not the other way around. More importantly, sex and gender are always racialized and classified, and vice versa. While elite white women were considered too delicate to work, poor, enslaved, and colonized women were required to toil away. Poor women, enslaved and colonized women whose labor was extracted, were thus not seen as “real women,” and deemed more androgynous, mannish, and animal-like (Lugones 2007, 2016). To talk about coherent and universal biological categories of sex, gender, race, or nation is to ignore the lessons of history.

Ideologies of sex and racial differences and their inter-relationships are powerfully evidenced in histories of eugenics—where elites (through the state and other mechanisms) controlled individual reproductive abilities and rights (Subramaniam 2014). What scholars in fSTS have powerfully shown is that these ideologies ground the terminologies of biology. Binary sex (male, female), binary gender (masculinity, femininity), and complementary heterosexuality are not biological ideas but ideologies (Lorber 1993; Lewontin 1996; Bleier 2010). It is the power of these ideologies masquerading as science that continues to shape the dominance of the west and those deemed elite: white, rich, straight, able-bodied, and male.

Conceptions of binary sex, gender, and sexuality are everywhere. What is particularly pernicious is the ways in which such ideologies promote gender essentialism, the belief that binary sex and gender are discrete and dichotomous categories and that they yield biologically determined, immutable properties of bodies. Sex and gender are not separate categories, but intimately interconnected. Men are masculine and women are feminine, and if popular culture will have it, they are relegated to two different planets—Mars and Venus! In such a view, no social programs that promote gender or racial justice can succeed since these are innate characteristics. For example, while much has changed thanks to social movements, foundational ideas that render men as active and rational and women as passive and emotional still endure. These ideas translate into a sociobiological phenomenon where men are celebrated as excellent in science and mathematics and women in the humanities and arts (Nürnberger et al. 2016). These phenomena in turn reinforce the gendered ideologies of men and women.

As we detail this later in the paper, these ideologies are embedded in scientific theories such as man the hunter and woman the gatherer (Longino and Doell 1983; Milam 2020). Darwinian logics of sexual selection shaped theories of anisogamy, ultimately leading to rules such as Bateman’s principle that promote a view of sexuality where males are promiscuous and females are coy (Roughgarden 2004; Tang-Martinez 2016), claims in animal behavior of alpha males and harems, or the infamous theory that rape is a biological adaptation (Hrdy and Bleier 1986; Bleier 2010). As we describe later, while feminists have thoroughly refuted these ideas (Tang-Martinez and Ryder 2005; Hrdy 2009; Gowaty et al. 2012; Tang-Martinez 2016), they endure. The idea that men and women are fundamentally different (as complements of each other) is deeply rooted in western culture. It is a huge leap to start from the observation of differences in gamete size, to then make claims that males and females are completely different in their bodies, physiologies, behaviors, intellect, and capabilities (Roughgarden 2004). Despite many critiques of claims of sexual (and racial) differences, these debunked ideas persist because they are not ideas but deeply rooted ideologies (Tang-Martinez and Ryder 2005; Hrdy 2009; Roberts 2014). This pattern is especially ironic given that the familiar vocabulary of male/female as universal, natural, biological, and essential categories is not an empirical observation but a definition. In general, the individual that produces the relatively smaller gamete is universally called the “male,” and the larger, the “female” (Garcia-Gonzalez et al. 2013), and the same definition applies to the female and male functions in hermaphrodites (Breed and Moore 2016). Yet, the science of “difference” is everywhere, shaping our lives in small and big ways. For example, it is striking that our understanding of female genitalia and their evolution remains largely a neglected area of study (Sloan and Simmons 2019).

When transferred to distantly related lineages like plants, these constructs of a sex/gender binary and gender essentialism can have harmful consequences. If plants, animals, and humans are all alike, surely, the argument goes, the categories must be natural, indeed universal? One of the powerful consequences of transferring human biology to plants and non-human animals is the normalization and naturalization of western colonial ideology as biology. Ideology thus gets naturalized into inviolable biological law. While activists have fought to challenge gender, race, and other regressive norms in human societies, the same ideologies are pervasive in plant and animal biology. It is thus imperative that we carefully examine the language, theories, and terminologies we use in the plant sciences. When terminology, and the theory embedded in this terminology, does not hold up to biological scrutiny, we must abandon it for more accurate language. Arguments that human analogies make plant biology more accessible, cute, sensational, or powerful are distractions and must be refuted and refused.

## The sexual lives of plants

Carolus Linnaeus (1707–1778), considered the founder of modern taxonomy, ushered in a binomial nomenclature system giving each organism a unique, two-word name consisting of the genus and species name (for example, *Homo sapiens* for humans). In 1735, he published the Systema Naturae, in which he organized all biological life into a hierarchical set of groups—species into genera, genera into orders, and orders into classes. For plants, his nomenclature was based on a sexual system of classification, where the number and position of the reproductive organs helped determine its classification (Taiz and Taiz 2017). He saw flowers as endowed with binary sex—male and female. Londa Schiebinger (1993) argues that not only were his plants sexed, “but they actually became human; more specifically they became husbands and wives.” To be a Linnaean taxonomist meant that the scientist had to believe in the sexual life of flowers (Browne 1989).

As Schiebinger (1993) argues, the scientific revolution and the revolution in sexuality and gender came together to elevate plant sexuality as a central focus of botany. Linnaeus gave primacy to plant sexuality in an era where the growing scientization of botany coincided with an ardent sexualization of plants. The Linnaean system, based on sexual difference that was read through the evolving lens of human sexual relations, came to embody the “laws of nature.” In so doing, Linnaeus brought traditional notions of gender hierarchies “whole cloth into science,” incorporating fundamental aspects of (western) human social order into frameworks to characterize plants and the natural world (Schiebinger 1993: 17). Flowering plants are at the center of his resplendent vision of plant sexuality. If legal heterosexual marriage was at the heart of plant life, then Linnaeus was thorough in placing “sex” at the heart of plant taxonomy—plants that reproduced through vegetative and clonal means were deemed asexual, and non-seed-bearing plants such as ferns, mosses, algae, and fungi were grouped together within Cryptogamia (plants that marry secretly). Successfully promoting his work globally, Linnaeus’s work was very influential (Fara 2004).

To understand the sexual vocabulary of the plant sciences, we begin with angiosperms, or flowering plants. We recognize that angiosperms are not the only plants that reproduce sexually, but since our understanding of sexual biology is overdetermined by angiosperm biology, we focus on them exclusively here. The term angiosperm is derived from the Greek words *angeion* (meaning vessel) and *sperma* (meaning seed)—plants with seeds derived from ovules contained in a protective vessel, unlike gymnosperms, where the seeds are derived from unprotected ovules. But angiosperm is itself a misnomer, and it should be Angio-ovulate and Gymno-ovulate, since what is consistently covered or naked at the time of pollination is the ovule (Tomlinson 2012). Whether it is the (colonial) human fascination for the spectacular that created a trade in flowers (de Klemm 1990) or an obsession with statistics of species diversity or numerical abundance, angiosperms have received considerable attention in the plant world. With over 350,000 species that represent ∼90% of land plant species, angiosperms are by far the most diverse extant lineage of multicellular land plants. As a successful group, they have emerged as the main model group to study the fundamentals of sexuality and reproduction (Soltis et al. 2018). Darwin called the abrupt origin and rapid diversification of the angiosperms “an abominable mystery,” and their diversity of forms remains a lively debate within the plant sciences (Friedman 2009). Is sexual reproduction, mediated by pollinators and dispersers, an adaptation that explains the astonishing variation of flowering plants? Or, are other forces more important drivers? The debate is far from settled, but the importance of sexual reproduction remains a popular theory to explain the diversity of angiosperms, including the evolution of their varied reproductive structures, mating systems, and their co-evolution with a wide range of biotically mediated engagements with other organisms, such as insect pollination and dispersal (Hernández-Hernández and Wiens 2020).

In endowing plants with human sex, Linnaeus developed an elaborate sexual vocabulary by blurring the differences between plants and humans (Bondestam 2016). He characterized plants by their reproductive parts—literal and elaborate analogies of convergence: the filaments of stamens were the vas deferens, the anthers were the testes, and pollen was the seminal fluid. In the female, the stigma was the vulva, the style the vagina, the pollen tube the fallopian tube, the pericarp the impregnated ovary, and the seeds the eggs. Others even argued that the nectar in plants was equivalent to mothers’ milk in humans (Schiebinger 1993). In his nomenclature, Linnaeus did not use the non-sexual terminology of pistil and stamen—but introduced *andria* and *gynia*, which are derived from Greek for husband (*aner*) and wife (*gyne*). This new terminology gave rise to a classification system where classes of plants end in “*andria*” (*monandria, diandria*, and so on) and orders in “*gynia*” (*monogynia, digynia*, etc.). Note that class (male) is ranked higher than order (female). In short, Schiebinger (1993) argues that “Linnaeus saw plants as having sex, in the fullest sense of the term” (23).

And so, the plant sciences came to inherit a sexual system modeled on “gentlemen and ladies” and their attendant botanical vocabularies of marriage: male, female, sex, gender, reproductive anatomies of male and female, sexual systems, breeding systems, and an imagined compulsory and complementary heterosexuality. We hasten to add that, while it is beyond the scope of this paper, such a binary model does in fact poorly capture human sexuality as well (Fausto Sterling 2000)! Plant reproductive biology enshrines a particular moment of colonial history where western sexuality emerged as scientific, natural, and universal (Pratt 2007). It is time to dismantle it and liberate it from its colonial histories.

## Plant reproductive biology

In placing binary sex (male/female) and heterosexuality as key attributes of plants, sexual reproduction has loomed large in the plant sciences, and with it, a focus on angiosperms. It is worth remembering that while angiosperms are indeed a diverse group of organisms, only 5–6% of angiosperms are dioecious (defined as having separate male and female plants) and 7% are monoecious (defined as having separate male and female flowers in the same plant) (Cronk 2022). The vast majority of angiosperms—over 85%—are “bisexual” (or hermaphroditic) with perfect flowers (defined as having both “male” and “female” parts or a complete set of all reproductive organs). Unlike humans, we should also note that many angiosperms can self-fertilize, again a significant difference ignored in the easy analogies of plant and human sex. We reflect on two sets of issues. First, broad issues in plant reproductive biology, and second, particular issues around terminology.

Glossary **Queer:** is perhaps best described by Eve Sedgwick: “That's one of the things that “queer” can refer to: the open mesh of possibilities, gaps, overlaps, dissonances and resonances, lapses and excesses of meaning when the constituent elements of anyone's gender, of anyone's sexuality aren't made (or can't be made) to signify monolithically” (Sedgwick 1993: 7).

**Naturecultures** is a term coined by Haraway and Goodeve (2013) and insists on an interdisciplinary approach to studying the world. Refusing binary worlds of nature and culture, naturecultures allows us to understand the entangled worlds of bacteria, viruses, fungi, plants, animals (and human) as more-than-human worlds.

**M reproduction:** when reproduction involves only mitosis as in vegetative reproduction.

**MM reproduction:** when reproduction as in most angiosperms involves mitosis and meiosis in the production of a new generation.

**Pistillate and Staminate:** flowers—refers to flowers that only produce ovules or pollen.

**Parts of the flower:**

- Staminate flowers have stamens, but no fertile pistils. Stamens are composed of filament and anthers. Fertile stamens produce pollen.
- Pistillate flowers include the pistil, but no fertile stamens. Pistils typically have an ovary, a stigma and a style, and are composed of one or more carpels. Fertile pistils produce ovules.
- Many flowers also have sepals, petals, and pedicels.

**Binate** signifies the presence of both stamens and pistils in a flower. In case where there is a preponderance of stamens or pistils, we suggest—Binate(s) and binate(p) to reflect that.

**Gamete** a cell

**Dispersed gametes and retained** gametes refer to gametes produced by the megagametophyte and microgametophyte, respectively. The dispersed gametes produced by the microgametophyte are called sperm cells. The retained gametes produced by the megagametophyte are called the central cell and the egg cell.

**Sex and Gender:** are related terms that have undergone considerable transformations over time. In early definitions, sex referred to attributes of the body, a person's biological maleness and femaleness and gender to the non-physiological (cultural) aspects of masculinity and femininity. Sex and gender were correlated: males were manly and females, feminine. For early feminists, the distinction proved useful to draw attention to misogynist cultural expectations of sexual bodies. After many decades of feminist scholarship, these early definitions no longer hold. Today, sex is not only a character of the body but is also cultural, and gender is both social and biological. We can no longer use these terms to represent separate and non-overlapping concepts (Lips 2020, Schiappa 2022).

**Plant Sex and Gender:** In the plant literature, the earlier definition is what is most prevalent: sex as body parts, and gender as social/sexual identity. However, as we argue in the article, given that most plants have binate flowers, neither sex nor gender are obvious categories. We suggest that we move away from a binary conception of sex and gender to understanding both as quantitative traits.

### Key issues in plant reproduction

One of the key concerns we confronted in this work is that the use of biological terminology across plants and animals implies an evolutionary connection—whether by homology or analogy. Yet, as we have argued throughout the history of botany, the same terminology is less about an evolutionary connection than a metaphorical one. The anatomy and process of sex in plants and animals seem similar not because of their biologies but because of a history of anthropomorphic and linguistic linkages. Untethered from animal worlds, we seek to re-center plants in this re-examination and reassessment of plant biology. In interrogating plant reproductive biology, we begin by highlighting some overall insights.

First, so much of plant biology, like plant development, has emerged in the “shadow” of animal biology “without a critical assessment on whether basic forms of plant and animal construction and development are really comparable” (Kaplan and Hageman 1991). We believe it is time to move plants out of the shadow and into their own unique spotlight. This paper honors this tradition. Second, we worry that the word sex is overburdened with meaning, making it imprecise, value-laden, and thoroughly anthropomorphic. To be sure, plants produce gametes, which produce zygotes and embryos. And yet, we must ask whether plants really have sex like humans? We suggest not. It is this overarching term that links human sex and plant sex that is at the heart of reducing plant sexuality to a human model. In short, a vast territory of attraction, attachment, desire, love, affective, emotive, and sexual dimensions that mark discussions of sexuality in humans/animals is entirely absent in vocabularies of plant biology. We discuss this absence later in the essay. How then do we recognize the inventive and adaptive life of plant reproductive biology without reducing it to human sexual frameworks? Here, we think the terminology of mitotic (read asexual) and meiotic (read sexual) reproduction is both biologically and linguistically helpful. Meiosis is an early innovation and an important feature of eukaryotes. While different branches of eukaryotes have further adapted and evolved complex reproductive cycles that incorporate meiosis as a key feature (Mirzaghaderi and Horandl 2016: 15), meiosis is often singled out as an important feature of eukaryotic sexual reproduction that explains the tremendous variation that produced “evolvable” mating systems and stability to the genome that has resulted in “spectacular outcomes” (Goodenough and Heitman 2014). Even when organisms self-fertilize, meiosis is distinctly different from mitotic reproduction. We suggest that we introduce the terminology of mitotic reproduction (M) and meiotic (MM), which always also involve mitosis, although there exists tremendous variation in how MM reproduction unfolds. This terminology, we argue, is more accurate and also inclusive of the tremendous variation we see in organisms across the plant and animal worlds.

Third, much of the floral vocabulary is grounded in the female, one that highlights female sexuality. For example, when measuring reproductive potential, ovules often come to stand in for the fecundity of the plant, even though the dispersed gametes in pollen grains also contribute to reproduction. This may, of course, be because of challenges tracking pollen. But the centering of the female as the primary reproducer has a profound and overdetermined impact on our theories of sexuality and reproduction (where females, women, and mothers come to stand in as key agents of reproduction). Terminology such as “mother” and “daughter” cells reinforce the same point. Today, when technologies can trace the dispersal and fertility of pollen, it is time to revisit our methods for more accurate measures of fitness components in plant biology. As we see later in our discussion of sexual selection, feminists studying animal behavior revolutionized the male-centered understanding of animal behavior once they followed the contribution of sperm. Fourth, the demography of contemporary angiosperms must be understood as a history of botanies of “desire” (Pollan 2002). Humans have profoundly altered landscapes, demography, and the evolution of plants. Large numbers of plants are used in agriculture and horticulture and have been cross-pollinated and profoundly modified over generations of plant breeding. Understanding the demography of plants through their engagement with pollinators, dispersal, and cultivating agents (such as wind, water, fire, bees, birds, mammals, and humans) should be a key factor in our fascination with plant reproductive biology. If we are true to history, plant biology emerges as a profoundly nature-cultural project.

### A closer look at eukaryotic reproduction (MM reproduction)

Plant reproductive biology poorly fits a model of binary sex, gender, and sexuality. Cronk (2022) provides an excellent summary and analysis of these differences. First, clonal plants challenge the idea that asexuality is an evolutionarily unsuccessful strategy. After all, many such species have successfully survived millennia. Further, clonal reproduction and dispersal are not homogeneous, but also inventive and adaptive. For example, some plants exhibit apogamy (in some ferns, the sporophyte develops from the gametophyte without fusion of gametes) and apomixis (where a plant produces asexual seeds). While beyond the scope of this paper, it is important to recognize the innovations of non-sexual reproduction in plants. Challenging the idea that sexual reproduction was selected for its production of variation, Cronk argues that sexual reproduction does not always generate recombinant and variable organisms. Conversely, mutation rates and selection in long-lived organisms that reproduce through clonal reproduction can generate substantial heritable variation.

**Fig. 1:**
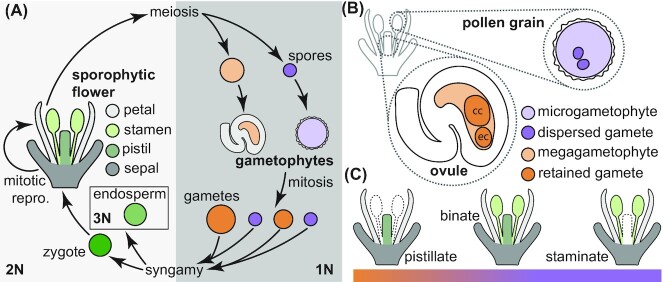
Angiosperm reproduction. (**A**) A generalized angiosperm lifecycle, which can include both mitotic (left) and meiotic (right) reproduction. (**B**) Ovules, produced in the carpels that comprise the pistil, and pollen grains, produced in the anthers of stamens, house gametophytes and gametes. Not all gametophyte cells or nuclei are shown. (**C**) Flowers occur along a quantitative gradient of either producing only ovules in carpels (pistillate), producing both ovules and pollen (binate), or producing only pollen in stamens (staminate).

Second, plant and animal gametes are not homologous and are derived from completely different processes in widely divergent life cycles. Land plants go through an alternation of generations—between sporophytic and gametophytic generations. The diploid sporophyte undergoes meiosis to produce haploid spores, which germinate and produce the haploid gametophyte and, in turn, the gametes. Angiosperms have three types of gametes: sperm cells, egg cells, and central cells. Sperm cells are produced by mitosis in microgametophytes enclosed in pollen grains; egg cells by mitosis in megagametophytes enclosed in ovules (Fig. 1). The mature “secondary nucleus” of the central cell is derived from the fusion of two mitotically derived “polar nuclei” and is thus usually diploid. One sperm cell fuses with the egg cell to form the zygote, and the second fuses with the central cell to form the endosperm that nourishes the growing embryo (Friedman and Ryerson 2009; Dresselhaus et al. 2016; Li and Yang 2020; Kaplan and Specht 2022). Through mitosis, the zygote develops into a diploid sporophyte, nourished by the (usually) triploid endosperm. In contrast, animals directly produce haploid gametes through meiosis. There is no multicellular haploid phase of a gametophyte. In addition, double fertilization is unique to the seed plants (Friedman and Floyd 2001). Likening plant gametes and angiosperm life cycles to animal models thus significantly misrepresents plant reproduction. Third, the germline is most often set apart early in animals. In contrast, plants are usually indeterminate in their growth and can produce new germline cells throughout their lives.

Finally, unlike animals, plants exhibit considerable environmental flexibility in morphological sexual expression. The degree and ratios of staminate and pistillate flowers can vary considerably within seasons, between populations, and across years (we prefer the terms staminate and pistillate, or carpellate, rather than pollenate and ovulate, which could accommodate gymnosperms, although either pair of terms is preferable to male and female). Such flexibility in sexuality should challenge any notion of a stable conception of gender or sexuality in plants (Whitehead et al. 2018).

In summary, during development, lineages of many flowering plants routinely subvert the “normal” reproductive process to produce considerable floral and sexual variation. Plant mating systems often vary widely between species and populations of the same species—from exclusive outcrossing to predominantly self-fertilization (Whitehead et al. 2018). Many plants have an eclectic combination of the above types in mixed mating systems—these systems have been shaped by evolution, ecology, changing environments, and varied pollination and dispersal mechanisms.

Contemporary botany thus remains—unfortunately—caught up in a Linnaean imagination. If we inherited a Linnaean system of plant nomenclature that ill represents the diversity of plant sexuality, how do we undo this, and what might we replace it with? While science can never be outside of culture or history, we can certainly pay greater attention to the biology of plants and be reflexive of the histories we have inherited (“reflexivity” has emerged as a term in the humanities that encourages a questioning of the process of research, in particular the many taken-for-granted assumptions we make in any discipline). From such a view, new possibilities emerge. One of the key sites is that of language, especially terminology in reproductive biology. We discuss the problems with these terms and suggest alternate terminology.

## Key terms in plant reproductive biology: naming and re-naming

Interrogating terminology in plant biology through scholarship in queer and gender studies is filled with irony and insight. Within contemporary biology and in mainstream culture, a binary sex/gender system is firmly in place, i.e., two biological sexes, male and female, who present their cultural phenotypes or genders as masculinity and femininity. The two sexes and genders are seen as complementary, leading to the normative condition of heterosexuality. Normative refers to phenomena that are culturally (and usually also scientifically) agreed upon as acceptable and desirable. In short, they are scientifically and socially accepted and valued. While this model is seen as natural and therefore biological, historians of sexuality remind us that this is a very recent story that we tell about human sexuality. In the early 20th century, the life sciences understood normal human sex to be bisexual, with every individual being a mix of male and female. Indeed, all (human?) life was believed to start out in utero with the potential to become any sex during development (Gill-Peterson 2021). Historians of medicine note the emergence of not only binary sex but binary bodies (sexed anatomies of skeletons, brains, and physiologies through hormones and other means) (Laqueur 1992). As Gagliano et al. (2017)insightfully suggest, perhaps the current story we tell about plants is part of a larger phenomenon of the “zoologizing” of botany—where ideas about animals (particularly humans) permeate our narratives about plants in an attempt to make them more interesting and accessible.

Further, recent scholarship on the histories of slavery and colonialism has challenged the very idea of a universal “sex” and “gender” even in human populations. Scholars argue that scientific constructs of sex and gender are in fact colonial constructions imposed throughout the world during the project of colonialism. Scholars from a range of disciplines—indigenous studies, decolonial studies, postcolonial studies, ethnic studies, Black studies, queer studies, and trans studies—critique enlightenment theories of sex and gender largely produced by white Anglophone scholars—helping us historicize extant terminologies about sexuality within the longer history of racial and violent colonial systems of slavery and conquest (Spillers 1987; Lugones 2007; Driskill 2016; Snorton 2017; Gill-Peterson 2021). It is significant that before colonial conquest, various other arrangements, vocabularies, and conceptions of sexuality existed among human societies world-wide. Just as Columbus claims to have “discovered” America, a land with thriving cultures of native people, western colonists claim to “discover” plants long used by peoples in colonial worlds and then consolidate this knowledge in the name of a universal “botany.” Knowledge within colonized cultures was both appropriated and its origins erased—but rarely acknowledged. Colonial science set in place infrastructures of knowledge, particularly a hierarchical view of sex, sexuality, race, and nation. These colonial ideologies eventually were routinized and made normal and natural through the power of western science (Fara 2004; Schiebinger and Swan 2007; Bataski et al. 2016).

## Floral morphology

Why rethink floral vocabulary? Our primary goal is to more accurately describe plant worlds and thus not limit the complexities of plant reproduction by analogizing it with human reproduction. Revisiting our vocabulary will produce better and more accurate plant sciences. We have tried to draw on history for this exercise, since before the “zoologizing” of botany, plants had a different set of vocabularies. Prior to Linnaeus, within medieval cosmology, plants were understood as useful to humans as food and medicines. As late as the Renaissance, botanists named parts we consider sexual today with non-sexual vocabularies—the stamen, from Latin, denoting the warp thread of fabric, and the pistil, the *pisl* because it resembled a pestle (Schiebinger 1993). We suggest that we return to these earlier terms when we talk about floral morphology. Here are the key terms we continue to use, with each term’s meaning in parenthesis—anthers (or flowery), carpel (or fruit), filament (or thread), ovule (or a little egg), pedicel (or foot), petal (or leaf), pistil (or pestle), pollen (or fine flour), sepal (or separate), stamen (or warp in the upright loom), stigma (or to mark), and style (or writing tool). We have purposefully alphabetized this list so as not to privilege the sexual or reinforce a binary reproductive system. We also suggest the term “binate” to signify the presence of both stamens and a pistil in flowers. In cases where plants regulate and shift their fertility to time their staminate and pistillate selves (Whitehead 2018), we could refer to them as binate (*p*) and binate (*o*) to signify a preponderance of staminate or pistillate flowers.

One term we examined carefully is the word gamete (and with it, gametophytic). The Oxford English Dictionary traces the term to the Greek “to marry.” However, the history of the term is very interesting. While erroneously attributed to Gregor Mendel, it was coined by Eduard Strasburger, then Professor of Botany at the University of Jena, and first used in the scientific literature in 1877 to describe these “mobile elements” in the single-celled green alga *Acetabularia*. The term describes the (haploid) cells, which in *Acetabularia* are isomorphic, that fuse to produce a zygote and contrasts the “zygote” with the “spore,” which Strasburger attributed to asexual reproductive cells (Crawley 1966; Mandoli 1998; Herranz 2012). Given its origins in the field of botany (rather than transplanted from the animal or human worlds), we suggest we retain the term gamete. Although the fusion of the central cell and sperm cell in angiosperms generates endosperm and not a zygote, we see three reasons to use the term gamete to include both the egg cell and the central cell: (1) The polar nuclei in the central cell and the egg cell are derived from the same series of mitotic cell divisions; (2) there is fusion of the central cell and the sperm cells, with fusion being one of the defining characteristics of gametes; and (3) the endosperm may have been evolutionarily derived from a second zygote. Indeed, the central cell is commonly referred to as a gamete in the field of cell biology (Friedman and Ryerson 2009; Dresselhaus et al. 2016; Li and Yang 2020; Kaplan and Specht 2022). We have also retained the terms micro- and mega-gametophyte since they do not carry a loaded history.

For the gametes produced by the mega- and micro-gametophytes, we suggest we move to the terminology of “dispersed gametes” and “retained gametes,” respectively (Fig. 1). Ideally, we would be able to dispense with the widely used and gendered terms of sperm (from Latin seed or semen, also used in the plant worlds) and egg (object laid by a female bird, again imported into botany). However, while “retained gametes” can substitute for the identical sperm cells, there are two kinds of retained gametes—the egg cell and the central cell. To avoid introducing too much terminology at once, we have retained the egg cell for the moment. This new terminology gets around the contentious and anthropomorphic concerns about gamete size and their inevitable anthropomorphizing into problematic suggestions of fidelity and harmful stereotypes of coy females and promiscuous males. This is most important because land plants have both retained and dispersed gametes—plants should not be a site for the reproduction of such misogynist tales and frameworks.

Cardoso et al. (2018) also point to the varied and confusing usage of key terms like “reproductive system,” “mating system,” and “sexual system” as synonyms even when used differently in the literature. We are mindful of this and, as they suggest, try to distinguish between sexual, floral, incompatibility, and mating systems, as well as apomictic systems. We wish to recenter plant and floral biology through their individual morphologies, anatomies, and physiologies—vegetative and reproductive—which helps highlight plants’ complex developmental pathways and distinctive life cycles, adapted to unique environmental and ecological contexts. Such vocabularies open up the possibilities of describing plant biology without the baggage of the politics of sex in contemporary understandings.

## Sex and gender

The terms sex and gender have been extensively adopted by plant biologists. However, we argue that these are not homologous or even analogous terms, phenomena, or morphological organs, but rather, in our view, a forced and unhelpful adoption. Unlike animals, the cells destined to become the gonads and produce gametes are not sequestered early in development to one location in plants. Instead, in most angiosperms, the flowers that will eventually house the gametophytes are usually produced iteratively over a long period of time. When a plant or flower produces gametes from pollen and ovules, their numbers can vary considerably. Not all ovules form seeds, and pollen disperses to other plants, often far away. Thus, the relative contributions of staminate and pistillate flowers can thus only be confirmed *posthoc*. Sex is not a character that is an intrinsic quality of most plants. It is a dynamic category—plants house dispersed and retained gametes that can vary dramatically. Some angiosperms reproduce largely through their pollen contributions, others exclusively through their ovules, and most through a combination of both. More importantly, these are not stable characteristics and can vary within and across seasons. The fitness of ovules and pollen is therefore dynamic and shifting (adaptations for physiological, ecological, or environmental reasons).

How do biologists adapt the terminology of sex and gender to plant life? The whole idea of sex determination, as we theorize it in humans, falls apart in the angiosperms. Because only gametophytes can produce gametes, plant sex determination is often assumed to be a gametophytic process. However, this is very much not the case in angiosperms. In angiosperms, sporophytic development determines whether carpels, stamens, or both will develop into flowers (Pannell 2017; Cronk 2022; Klein et al. 2022). The identity of carpels vs. stamens determines what the products of meiosis will be and, in turn, what kind of gametes will be formed (retained vs. dispersed). This means that although flowers cannot produce gametes or have sex, floral development is critical to angiosperm sex determination. In addition, systems with separate pistillate and staminate plants (dioecy) and sex chromosomes have evolved repeatedly and relatively recently in the angiosperms (Charlesworth 2002). More significantly, plant reproductive systems are expressed in the individual and vary widely among individuals of the same species within populations (Whitehead et al. 2018). These and many other reproductive system adaptations produce astonishing variation and flexibility that shows little resemblance to animal reproduction (Barrett 1998).

Plant gender refers to the relative representation of stamens and carpels, or phenotypic gender, and the relative contribution of genetic material to the next generation through pollen and ovules, or functional gender (Lloyd 1980). The distribution of phenotypic gender and functional gender can change from season to season, and year to year. Plant gender is often regarded as a plastic and quantitative trait (Pannell 2017). The term “gender” has also been invoked in the case of organisms that exhibit male sterility who are fertile only through their ovules, even though their anatomies may include infertile anthers (Mazer et al. 1999).

On close examination, the imposition of the terms sex and gender, invented more recently for human worlds, and those imposed on plant worlds are not in the least analogous. Neither sex nor gender are equivalent in animal/human biology. Instead, some plant biologists have introduced the terms quantitative sex (Faux and Berhin 2014; Faux et al. 2014) and quantitative gender (Lloyd 1980; Borges 1998) to better represent the complexity of plant reproduction.

Recently, Martine (2023) has argued that sex is a biological category and gender a social category. Arguing that gender as a cultural manifestation is ill suited for plant worlds, he suggests we should dispense with the concept of plant gender entirely and only refer to plant mating systems purely in terms of sex. But is culture, or perhaps the environment or ecology of plants, an entirely inappropriate concept? After all, plant biologists have documented variation in mating systems among individuals of the same species across populations. Plant sex or gender is not an innate characteristic of a species but one that varies depending on local ecologies such as the density and frequency of individuals of the species or the availability of pollinating agents. In addition, plant developmental biologists have described the various developmental mutations and switches that allow for a rather flexible and dynamic model of sexuality as opposed to what a binary sex model proposes. However, given how ecological and environmental contexts shape population differences in the ratios of staminate and pistillate flowers, we wonder if “culture” or gender, while inappropriate—maybe in fact be a more appropriate term than sex? While we applaud Martine’s thoughtful commentary and analysis, drawing on recent work in feminist and queer studies, we would caution on an easy binary between biology/social or nature/culture. Indeed, scholars would argue that both categories of sex and gender are constructed terms—deployed for particular political, biological, and social purposes (Lips 2020; Schiappa 2022). Given the proliferation of queer movements across the world and the celebration of cultures that house more than two sexes and genders, both sex and gender have been expanded beyond the binary of male/female. Given this, we would suggest dispensing with both categories of sex and gender—or at least understanding them both in their quantitative dimensions. If we only retain sex, it will further ossify biologically determinist ideas of “true” and natural sex. Our preference would be to embrace terms (some already in use and some that need invention) that help us better describe plant biology, such as quantitative mating systems, monoecy, dioecy, facultative and obligate outcrossers and selfers, staminate and pistillate flowers, all modified through incompatibility systems and mutations in developmental genes.

In addition to dropping the vocabulary of male and female as well as sex and gender, we also need to rework the language of husbands and wives that are enshrined in botanical vocabularies such as andro-dioecy or gyno-monecy. The andro- and gyno- are prefixes for an astonishing array of terminology to capture plant variation in floral permutations and combinations of staminate and pistillate flowers. We need to move away from the language of matrimony for plants.

## Sexual reproduction

The categories of sex and gender and the vocabulary around reproduction are key to theories of sexual reproduction, especially in animals. A central argument of this paper is that the immense intellectual infrastructure surrounding sexual reproduction in animals has been imported—and not very usefully—into the plant world. Originating with Charles Darwin, theories of sexual selection, in particular, loom large in the politics of gender and race (Richards 2017). In attempting to explain traits that don’t appear to increase the longevity or fecundity of an organism, avoiding the actions of natural selection, Darwin developed the theory of sexual selection, which has been intensely debated for more than a century and a half (Shuker and Kvarnemo 2021). Indeed, biologists are still trying to adequately define the “grey zones” of sexual selection, especially as they expand the scope of Darwin’s original formulation to include an account of female sexuality (Alonzo and Servedio 2019; Shuker and Kvarnemo 2021).

Darwin proposed sexual reproduction also as a “testing ground of racial character” and “a causal force that could create new races” (Sheldon 2021). Sexual selection, while influential, has had an uneven history. In developing this theory, Darwin argued for male–male competition and for female choice, endowing female animals with agency. He naturalized female choice in animals but, in contrast, normalized male choice among humans, creating a tension that ultimately threatened the success of his theory. “Misogyny,” as Rosenthal and Ryan (2022: 375) argue, “infiltrated his understanding of human mating patterns and polluted his general notion of the value of women in society.” Other biologists subsumed sexual selection within natural selection. Indeed, Evelleen (2017)detailed history of sexual selection argues that by the turn of the century, sexual selection was all but dead. Thomas Hunt Morgan concludes: “The theory meets with fatal objections at every turn” (quoted in Richards 2017: 526).

During the Modern Synthesis, there was little development of sexual selection except in the context of speciation. It was only in the 1970s that the idea sprang back to life. Erika Milam (2010) calls this narrative of neglect in the ensuing period the “eclipse narrative” of the history of sexual selection (one which Milam contests). With the emergence of human sociobiology in the 1970s and 1980s, leading biologists such as Trivers (1972), Wilson (1975), and Dawkins (1976) reinterpreted sexual selection within modes of rationalist and individualist game theory and promoted it through biological theory in the new sociobiology, where it soon became a dominant paradigm (Richards 2017). It is within this context that feminist critics, especially a growing number of women scientists, challenged the male-centered narrative of the new sociobiology, leading to a conflict between sociobiologists and feminists. Some feminists in turn developed a “feminist sociobiology,” where sexist ideas of males and females were reworked into models where females were more empowered. Theories such as cryptic female choice highlight female-driven morphological, behavioral, and physiological mechanisms (Eberhard 1996; Firman et al. 2017). But many other feminists continue to insist on a fundamental incompatibility and “irreconcilable differences” between feminism and sociobiology (Tang-Martinez 1997; Hoquet 2010).

Of particular note is the pushback on the very idea of sexual selection. Joan Roughgarden (2004, 2005, 2009), for example, argues that our view of animal behavior will be enhanced if we abandon the idea of sexual selection altogether and instead understand the fitness of organisms through “social selection,” as opposed to a singular focus on sexual selection. The sociality of organisms can have adaptive functions—helping build trust, cooperation, mutual aid, play, and pleasure, all of which can be important for survival and the fitness of the organism.

We narrate this abbreviated history of sexual selection to highlight both how the histories of race and sex shaped the histories of sexual selection, but also how feminist responses to these questions remain alive, multiple, and contentious. Nonetheless, theories of sexual selection have been long imported into the plant sciences (Anderson and Iwasa 1996; Skogsmyr and Lankinen 2002; Moore and Pannel 2011). The variability of plant sexuality should give us deep pause on relying on vocabularies of a binary sex/gender system as natural, normal, or normative. Within plant worlds—where the vast majority of flowering plants are not only both staminated and pistillate, but also shift ratios between the two and vary the timing of fertility—genetic variation and environmental differences together produce a breathtaking array of differences. Complicating the categories of sex, gender, and sexuality allows us to describe the biology of plants through the language of plants rather than human-imposed sexual scripts. It opens up the possibility of not privileging the “sexual” as the only aspect of a plant that is important from the point of view of evolution. Finally, it allows botany to present the complexities of morphological arrangements, mixed mating systems, genetic mutations and variations, and plant incompatibility systems to the public. This challenges the stronghold of western and colonial narratives of a binary sex of “gentlemen and ladies” to highlight the exuberance of plant sexuality.

Finally, what is particularly unfortunate in imposing human and animal frameworks onto plants is how this ignores that plant reproduction is often mediated through other creatures (insects, birds, mammals) and the elements (wind, water, air, gravity). For example, orchids display a breathtaking variety of plant-animal interactions filled with drama-filled stories of mimicry, deception, pseudo-copulation, rotting flesh, and even death. Enforcing human-centric vocabulary when studying non-human reproduction severely impedes our ability to open up new botanical scientific vistas and possibilities. Reducing the extraordinary variation of multi-species ecologies into the limited possibilities of binary sex reveals not only the dangers of poor science, but also a singular lack of our imagination!

## Re-imagining reproduction

In conclusion, we will admit that moving away from male and female is not easy. Years of cultural education have made such binary thinking accessible and ingrained in our conceptual vocabularies. But it is precisely for these reasons that so much cultural work gets done—the unexamined use of old terms because it is easy and ingrained reinforces of traditional roles and stories about males and females. When we tell the same stories about plant worlds, we not only misrepresent plants, but we reinforce patriarchal and colonial notions of humans. At first, staminate and pistillate did seem like a mouthful—but with time, it got easier. Anti-normative vocabularies are always difficult because they challenge established patterns—but they are both necessary and valuable. In this essay, we’ve taken a very small step in addressing what we see as some of the key problematic terms. Perhaps through such small steps, we can rework our terminologies of plant biology.

In working on this project, we went through several biology textbooks to track the usage of vocabulary. It is apparent that even the most well-meaning biologists, we think, end up using male/female or sex/gender as a shorthand for accessibility. Understanding the complex biologies of plants in the binary vocabularies of normative human sexuality makes memory easier, especially for a student reader of a textbook. The student reader, ultimately, understands plants as humans (with caveats of some exceptional facts). But, as always, here is the catch: it is accessible because it has been “simplified.” Simplified essentially means that we have taken away the vital exuberance, astonishing evolutions, and nimble developmental systems of plants. In addition, as plant biologists who are feminists, we think plants open up new and interesting possibilities to reimagine and reframe sexuality and reproduction. Though plant biology is limited, we can produce new vocabularies of reproduction that move beyond colonial archetypes of western sexuality. Plants provide fertile ground for us to begin to reimagine reproduction as a capacious phenomenon. Most importantly, recent work on horizontal gene transfer seriously brings into question the roles of transfer of genes across species rather than down the generations within species. These transfers may have profoundly and fundamentally shaped plant and animal evolutions (Ma et al. 2022). We must seriously consider the fact that the tales we tell about sexual reproduction, about the centrality of binary sex, gender, and sexuality, are convenient stories that continue to shore up not only particular biological worldviews but also colonial structures of power—of gender, race, class, and nation. This is an opportunity to rethink and reimagine plant biology while acknowledging and attending to the complexities of botanical histories. Rather than represent plants in a human image, we suggest that we describe plant reproductive biologies accurately, and plants’ exuberance may give us new vocabularies and imaginations for imagining less racist, misogynist, and transphobic worlds.

## Notes

From the symposium “Sexual diversity and variation” presented at the annual meeting of the Society for Integrative and Comparative Biology virtual annual meeting, January 16–March 31, 2023.
